# Automated Conformational Clustering Reveals Desolvation Mechanisms in a Wide‐Temperature‐Range Sodium‐Ion Electrolytes

**DOI:** 10.1002/advs.78718

**Published:** 2026-11-09

**Authors:** Long Huang, Kun Gao, Yiyao Han, Jing Yang

**Affiliations:** ^1^ School of Chemical Engineering and Technology Sun Yat‐sen University Zhuhai Guangdong People's Republic of China

**Keywords:** density‐based clustering, desolvation kinetics, molecular dynamics, sodium‐ion batteries, solvation structure, unsupervised machine learning

## Abstract

The desolvation kinetics of Na^+^ at the electrode‐electrolyte interface critically limit the wide‐temperature performance of sodium‐ion batteries (SIBs). Conventional radial distribution function analyses, which rely on spherical symmetry averaging, inherently fail to capture the microscopic conformational inhomogeneity that governs this process. Herein, we develop an automated conformational clustering framework that integrates unsupervised machine learning with a multiscale DFT (Density Functional Theory) MD (Molecular Dynamics) approach to resolve the temperature‐dependent solvation evolution in a model carbonate‐based sodium‐ion electrolyte. By mapping high‐dimensional trajectory data, this approach quantitatively identifies solvent‐separated ion pairs (SSIP) and contact ion pairs (CIP) conformations, revealing a temperature‐driven population shift from SSIP‐dominated (79.9% at 233.15 K) to CIP‐dominated (61.7% at 333.15 K). Density functional theory vertical energy calculations demonstrate that elevated temperatures substantially lower the SSIP‐to‐CIP energy penalty (from 98.3 to 47.0 kcal/mol). Consequently, the increased CIP proportion reduces the system‐averaged desolvation binding energy from 72.6 to 67.6 kcal/mol, which directly accounts for the order‐of‐magnitude enhancement in sodium cation mobility observed in molecular dynamics simulations. This work establishes a quantitative mechanistic closed loop linking microscopic conformational transitions to macroscopic transport kinetics, providing explicit molecular design principles for advanced wide‐temperature SIB electrolytes.

## Graphical abstract

Temperature‐driven solvation conformational shift reduces the desolvation barrier and enhances sodium‐ion mobility.

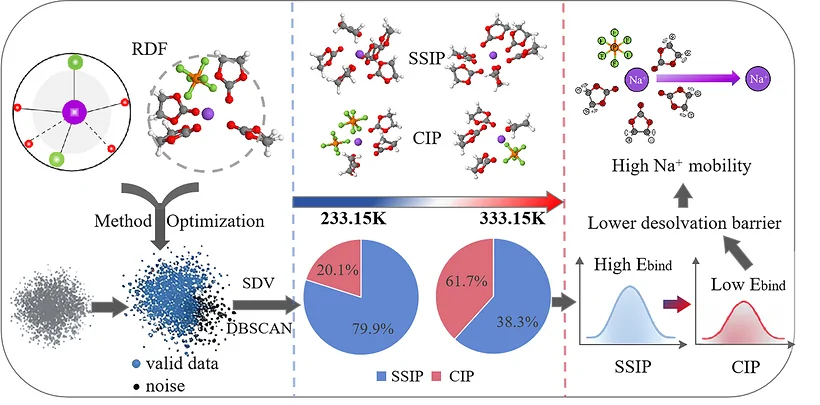

## 1 Introduction

Driven by the urgent demand for large‐scale energy storage, sodium‐ion batteries (SIBs) have emerged as a compelling alternative to lithium‐ion batteries (LIBs). Unlike LIBs, which suffer from the uneven geographical distribution and volatile pricing of lithium resources, SIBs offer abundant raw materials and cost‐effectiveness, making them ideal for grid‐scale applications [1, 2]. However, the larger ionic radius of Na^+^ (1.02 Å) relative to Li^+^ (0.76 Å) significantly impairs intercalation kinetics and ion transport rates [3]. This limitation is particularly pronounced under wide‐temperature operating conditions (−40°C to 60°C), presenting a critical bottleneck for the industrial deployment of SIBs [4].

As the medium for ion transport in batteries, the electrolyte directly determines the energy density, cycle life, and safety performance of the entire cell [5]. Recent studies have elucidated the underlying mechanisms: theoretical work has revealed the parasitic decomposition of carbonate solvents on P2–Na_2_/_3_Ni_1_/_3_Mn_2_/_3_O_2_ cathode surfaces [6], high‐valence Te(VI) doping has been shown to enhance carbonate stability on honeycomb layered oxides [7], and localized high‐concentration electrolytes have been designed to simultaneously improve oxidative stability and ionic conductivity [8]. These macroscopic advances underscore that electrolyte optimization is rooted in the precise regulation of microscopic solvation structures. For wide‐temperature applications, the desolvation of Na^+^, governed by the microscopic solvation structure of the electrolyte [4, 5] at the electrode‐electrolyte interface, is the rate‐determining step that limits battery performance [9]. In typical ethylene carbonate (EC)‐based NaPF_6_ electrolytes, the first solvation shell of Na^+^ generally exists in either solvent‐separated ion pairs (SSIP) or contact ion pairs (CIP) configurations. The coordination equilibrium of these complexes is quite temperature‐sensitive [10, 11, 12], such that the weak coordination bonds between Na^+^ and EC （Ethylene Carbonate） are readily disrupted by thermal motion, while the strong electrostatic interactions among charged species are relatively enhanced at elevated temperatures. This promotes anion penetration into the first solvation shell and the formation of CIP configurations [13, 14]. However, the precise temperature‐responsive evolution mechanism of these conformations remains elusive.

Even though molecular dynamics (MD) simulation is a powerful tool for investigating the solvation dynamics [15, 16, 17], conventional analytical methods exhibit significant limitations. The radial distribution function (RDF) and coordination number (CN) analyses rely on spherical symmetry averaging, which inevitably obscures the microscopic conformational inhomogeneity and transient states critical to kinetic processes. Specifically, RDF cannot effectively distinguish different geometric arrangements with the same coordination number (e.g., SSIP vs. CIP configurations), nor can it capture low‐probability transient conformations that play critical roles in kinetic processes, while enhanced sampling techniques suffer from high computational costs, poor universality, and reproducibility challenges [18, 19, 20, 21].

To overcome these challenges, this study proposes an automated analytical paradigm that integrates sorted distance vector (SDV) feature engineering, density‐based spatial clustering (DBSCAN), and density functional theory (DFT) calculations. Using a 0.1 mol/L NaPF_6_/EC dilute solution as a model system, we systematically investigate the conformational evolution of the Na^+^ first solvation shell across a wide temperature range (233.15–333.15 K). This multiscale framework quantitatively resolves the temperature‐driven population shift between solvent‐separated and contact ion pairs, and elucidates the underlying microscopic mechanism governing conformational transitions and desolvation kinetics from an energetic perspective, providing a robust theoretical tool for the rational design of wide‐temperature sodium‐ion battery electrolytes. The analysis presented here builds on a growing body of work that brings unsupervised, descriptor‐based methods to bear on electrolyte structure. SOAP descriptors combined with principal‐component analysis have proven effective for mapping the local environments of ions and correlating them with reductive stability in super‐concentrated electrolytes [22], and coordination‐based cutoff clustering has been employed to census coordination species and ion aggregates and to relate them to transport in sodium polymer electrolytes [23]. These studies establish data‐driven partitioning as a rigorous lens on solvation structure; the present framework advances this line toward a specific objective: the frame‐resolved quantification of coordination populations and their direct coupling to desolvation energetics and transport. The SDV descriptor encodes the coordination chemistry explicitly, with its dimensionality fixed by the physical coordination capacity (10 O + 6 F) and with permutation and rotational invariance, and DBSCAN is applied frame by frame in this descriptor space, returning absolute SSIP/CIP populations and resolving high‐energy transition states as explicit noise. Each conformational basin is then carried through to its DFT vertical desolvation energy and to the measured ionic mobility, closing a population–energy–transport loop. Looking ahead, coupling this physically anchored representation with higher‐dimensional descriptors such as SOAP （Smooth Overlap of Atomic Positions） and with machine‐learning potentials will carry the same closed chain to concentrated and multi‐component electrolytes, where the description of ion aggregation becomes essential.

## 2 Computational Methods

### 2.1 Molecular Dynamics Simulations

All molecular dynamics (MD) simulations were performed using the LAMMPS (version 2 Aug 2023) [24] software package with the OPLS‐AA all‐atom force field [25]. To isolate the intrinsic solvation behavior of an ideal dilute solution system (free from the complicating effects of ion aggregation and many‐body interactions prevalent in concentrated electrolytes), we constructed a cubic simulation box containing 1500 ethylene carbonate (EC) molecules and 10 NaPF_6_ ion pairs, yielding a precise Na^+^ concentration of 0.1 mol/L as a model system. The initial configuration was generated using the Packmol software [26], which ensures no atomic overlap and a uniform random spatial distribution of all species within a cubic box with an initial edge length of approximately 55 Å (Figure 1). A comprehensive summary of the key MD simulation parameters, including system composition, ensemble settings, and integration details, is provided in Table S1.

**FIGURE 1:**
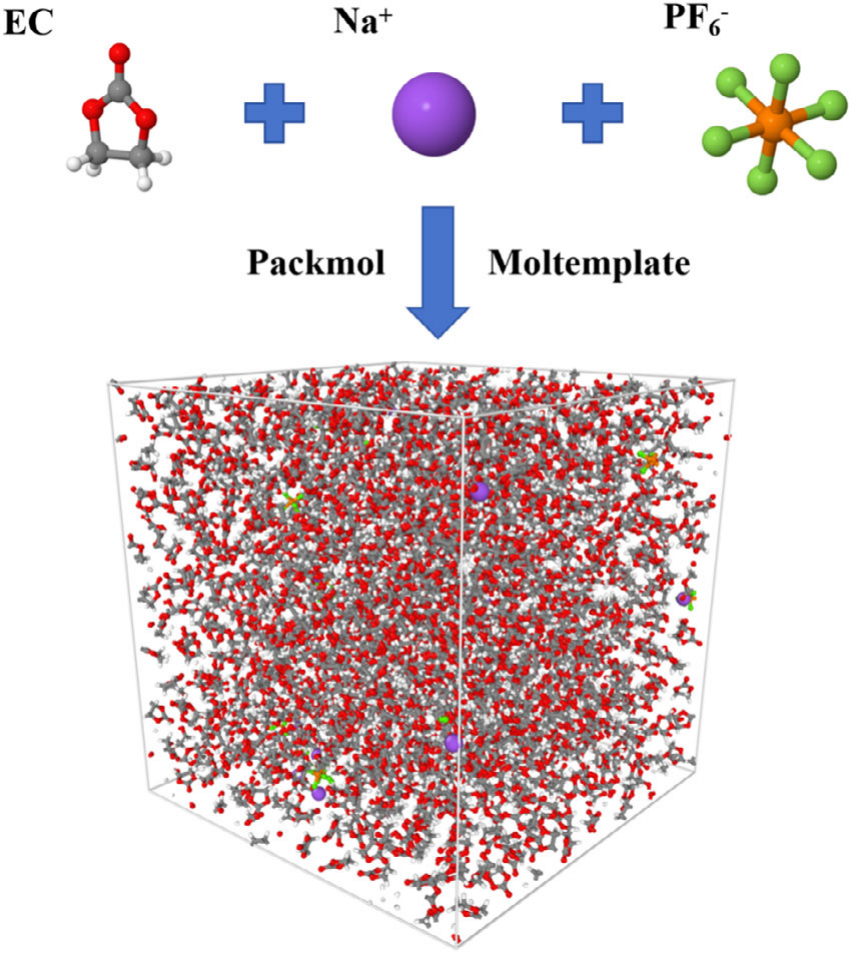
Initial simulation configuration of the 0.1 M NaPF_6_/EC electrolyte. The cubic simulation box contains 1500 EC molecules and 10 NaPF_6_ ion pairs. In the molecular representation, O, C, H, Na, F, and P atoms are depicted as red, gray, white, purple, green, and orange spheres, respectively.

The simulation protocol proceeded in three sequential stages. First, energy minimization was performed using the conjugate gradient algorithm with a convergence threshold of 1.0 × 10^−8^ eV/Å to eliminate any energetically unfavorable atomic overlaps. This was followed by a 5 ns equilibration run in the isothermal‐isobaric (NPT) ensemble to allow the system density to converge to its equilibrium value at each target temperature (Figures S1–S3). Finally, a 20 ns production run was conducted in the canonical (NVT) ensemble with a time step of 0.5 fs. Temperature and pressure were regulated using the Nosé‐Hoover thermostat and the Parrinello‐Rahman barostat, respectively. Long‐range electrostatic interactions were calculated using the particle‐particle particle‐mesh (PPPM) algorithm with a precision of 1.0 × 10^−5^, while van der Waals interactions were truncated at a cutoff radius of 12.0 Å. Independent simulations were performed at three characteristic temperatures spanning the wide operating range of practical interest: 233.15 K (−40°C), 273.15 K (0°C), and 333.15 K (60°C). Because pure EC melts at about 36°C–37°C, the 233.15 and 273.15 K states should be understood as metastable supercooled liquids rather than equilibrium phases. EC supercools readily, and homogeneous nucleation is kinetically inaccessible on the nanosecond timescale and within a roughly 55 Å box; consistently, no crystalline order developed in any run, and the density and mean‐square‐displacement traces remained smooth and liquid‐like (density, Figures S1–S3; MSD, Figure 6a–c). Practical sodium‐ion electrolytes further suppress solidification by blending EC with low‐melting co‐solvents [27]. We therefore treat the low‐temperature simulations as a model of the supercooled liquid state and interpret only the temperature‐dependent trends, not the absolute phase behavior. Trajectory frames were saved every 2.5 ps, generating 8000 frames per temperature for detailed post‐processing analysis.

The force field parameters were rigorously validated for the current system. Parameters for ethylene carbonate (EC) molecules were obtained from the LigParGen web server [28, 29, 30], while those for Na^+^ and PF_6_^−^ ions were adopted from the work of Brian et al. [31]. This parameter set has been extensively benchmarked against experimental data and widely used in solvation structure investigations of carbonate‐based electrolytes, ensuring accurate predictions of ionic coordination environments and transport properties. As a direct check on the present system, our NPT equilibrium densities (1.3628, 1.3197 and 1.2546 g cm^−3^ at 233.15, 273.15 and 33Fast Fourier Transform3.15 K) agree within about 3% with the experimental density of liquid and supercooled EC (about 1.32 g cm^−3^ near the melting point and about 1.338 g cm^−3^ for supercooled EC at 25°C) [27]. The equilibrium freezing transition is not a variable in the present analysis, as our conclusions rest on relative temperature‑dependent trends within the liquid state. Quantitative treatment of the phase transition can be incorporated in future work via two‑phase coexistence or free‑energy calculations without altering the core SDV–DBSCAN analytical pipeline Detailed force field parameter sources, partial charge assignment schemes, and mixing rules are documented in Section S1.1.

### 2.2 Trajectory Post‐Processing and Solvation Feature Extraction

All raw MD trajectories were post‐processed using a custom automated workflow developed in Python based on the MDAnalysis library [32, 33]. The core preprocessing steps included molecular topology reconstruction and periodic boundary condition (PBC) unwrapping, as illustrated in Figure 2a.

**FIGURE 2:**
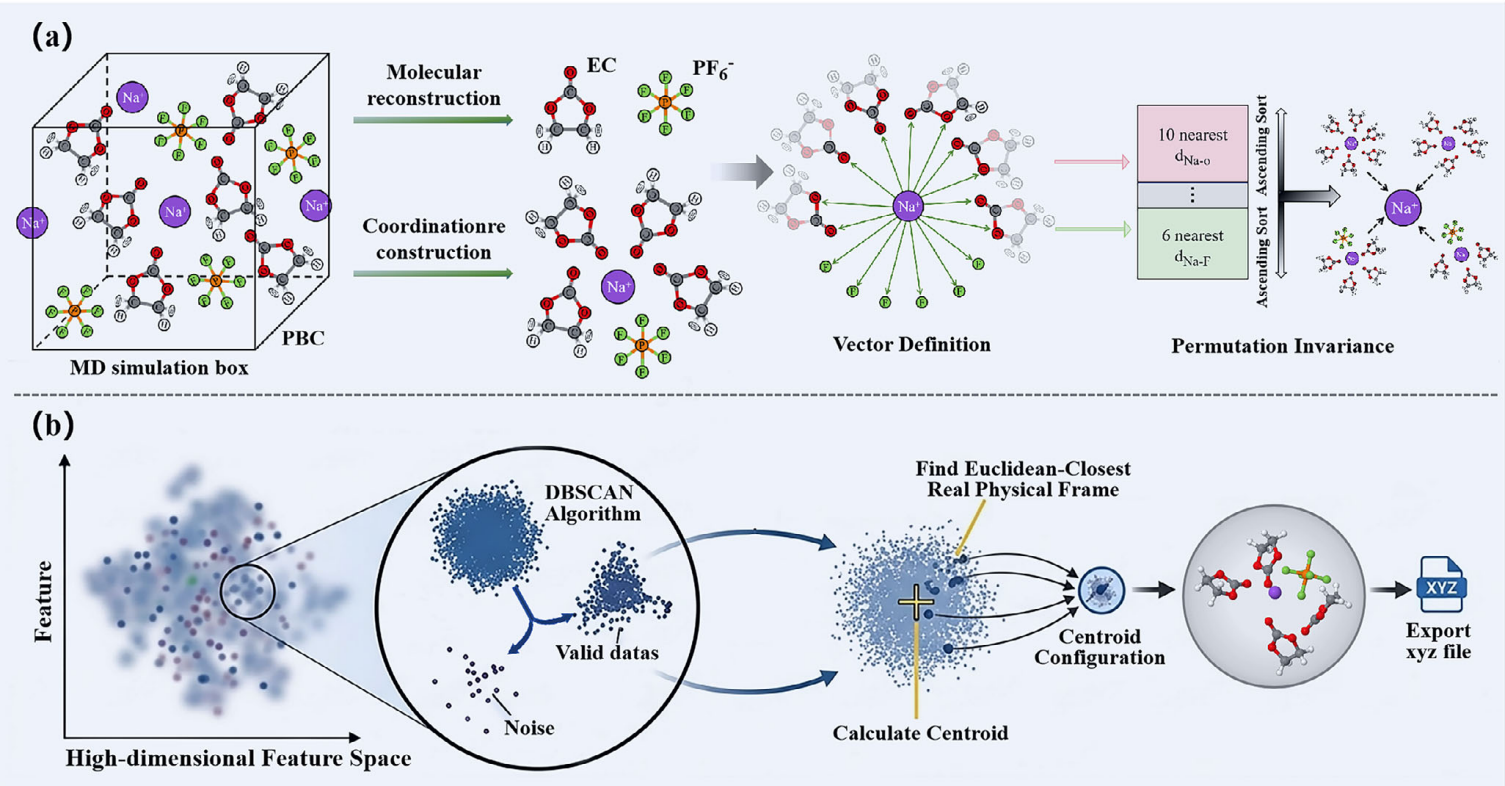
Schematic workflow of the automated SDV‐DBSCAN post‐processing framework. (a) Trajectory correction via molecular topology reconstruction (based on the BFS algorithm) and periodic boundary condition (PBC) unwrapping to ensure the accuracy of physical coordinates. (b) Inputting the permutationally invariant sorted distance vector (SDV) descriptors into the DBSCAN density clustering algorithm for unsupervised classification of solvation structures, thereby objectively identifying representative conformations (e.g., SSIP and CIP) and filtering transient noise.

Molecular topology reconstruction was performed using a breadth‐first search (BFS) algorithm to accurately identify intact molecular configurations. For EC molecules, the carbonyl oxygen atom was used as the anchor, and all covalently bonded atoms were identified by traversing the connectivity graph with a bond length threshold of 1.7 Å. For PF_6_^−^ anions, the central phosphorus atom was used as the reference, and its six nearest‐neighbor fluorine atoms were assigned to the same ion. This approach effectively eliminates the topological fragmentation artifacts that arise when molecules cross periodic boundaries.

To overcome the inherent limitations of spherical symmetry in conventional RDF analysis, we developed a sorted distance vector (SDV) feature engineering approach to quantitatively characterize instantaneous solvation conformations. For each Na^+^ in every trajectory frame, we calculated the distances to all surrounding oxygen atoms (both carbonyl O_c_ and ether O_e_) from EC molecules and fluorine atoms from PF_6_^−^. The oxygen and fluorine distances to Na^+^ were sorted separately in ascending order, and the first 10 oxygen distances and first 6 fluorine distances were concatenated to form a 16‐dimensional feature vector:

$$\mathrm{SDV}=[d\;O_{1},d\;O_{2},\text{…},d\;O_{10},d\;F_{1},d\;F_{2},\text{…},d\;F_{6}]$$

This descriptor exhibits inherent permutational and rotational invariance, providing a unique and compact mathematical representation of each instantaneous solvation conformation. This eliminates the ambiguity associated with coordinate‐system‐dependent descriptors and provides a robust foundation for subsequent unsupervised machine‐learning analysis. The rigorous mathematical definitions and proofs of the permutation and rotational/translational invariance of the SDV descriptor are detailed in Section S2.

### 2.3 DBSCAN Clustering and Conformational Classification

The SDV vectors from all trajectory frames were used as high‐dimensional data points for unsupervised clustering using the density‐based spatial clustering of applications with noise (DBSCAN) algorithm [34], as shown in Figure 2b. The DBSCAN algorithm was selected for this study due to three key advantages that are particularly well‐suited for solvation structure analysis: (1) it does not require a predefined number of clusters, eliminating the subjective bias associated with methods such as k‐means; (2) it can automatically identify cluster structures of arbitrary shapes; and (3) it effectively distinguishes and filters out low‐density, high‐energy transient “noise” conformations that do not represent thermodynamically stable states. In this work, the noise fraction was consistently less than 1% across all temperatures, confirming the dominance of well‐defined stable solvation conformations.

The key clustering parameters—the neighborhood radius ε and the minimum number of neighbors required to define a core point (MinPts)—were systematically optimized to ensure the statistical robustness of the results. After parameter tuning, ε was set to 0.8–1.0 Å and MinPts to 100 for all simulations. Each identified cluster corresponds to a statistically distinct and thermodynamically stable solvation conformation. The algorithmic kernel of DBSCAN, along with the physical justification for the systematic optimization of the ε and MinPts parameters, is elaborated in Section S3.

### 2.4 Representative Conformation Extraction and Density Functional Theory Calculations

To obtain high‐fidelity explicit solvation models for quantum chemical calculations, we developed a “centroid minimum distance method” to extract statistically representative conformations from each DBSCAN cluster. Specifically, we first calculated the geometric centroid of all SDV feature vectors within a given cluster. The actual MD trajectory frame whose SDV vector had the shortest Euclidean distance to this centroid was then selected as the representative structure for that cluster. This approach ensures that the selected conformation captures the average topological features of the cluster while strictly preserving the physical validity of atomic coordinates. Unlike direct coordinate averaging, which often yields unphysical structures with distorted bond lengths and broken molecular topologies in flexible solvation shells, selecting an actual MD snapshot (the medoid in feature space) inherently guarantees that all interatomic distances, bond angles, and molecular connectivity remain physically realistic and consistent with the force field.

When constructing the initial models for density functional theory (DFT) calculations, the central Na^+^ ion was placed at the origin, and a cutoff radius was defined based on the first minimum of the Na^+^‐O radial distribution function. To eliminate boundary effects induced by cluster truncation and preserve the electronic structure integrity of the solvation shell, we implemented a strict “molecular‐level integrity truncation strategy”: an entire intact molecule or ion was included in the DFT model if and only if at least one of its coordinating atoms fell within the cutoff radius. This approach fundamentally avoids the dangling‐bond artifacts and spurious boundary charge polarization that plague conventional atom‐level hard truncation methods.

All quantum chemical calculations were performed using the ORCA 6.0.1 software package [35, 36, 37]. The B3LYP [38, 39] exchange‐correlation functional with D3(BJ) [40, 41] dispersion correction was used in combination with the 6–31G(d,p) [42, 43] basis set, and the self‐consistent field (SCF) convergence criterion was set to TightSCF (1.0 × 10^−8^ Hartree). To preserve the temperature‐dependent thermal fluctuation characteristics and instantaneous topological structures captured by the MD simulations, we deliberately avoided conventional 0 K geometry optimizations and instead adopted a vertical single‐point energy calculation strategy. Specifically, the extracted representative MD snapshots were subjected directly to single‐point energy calculations using the conductor‐like polarizable continuum model (CPCM), with the dielectric constant of EC set to 89.0. The energies obtained from this approach represent the vertical binding energies of the system at the specific temperature of the corresponding MD simulation. The detailed DFT computational setup, including the ORCA keyword line and the physical justification for employing the room‐temperature CPCM dielectric constant across all simulated temperatures, is provided in Section S4. While unrelaxed snapshots inherently contain local thermal strain energy, these systematic errors cancel out quantitatively when calculating relative energy differences (ΔE) between structurally similar solvation conformations, ensuring the validity of our energetic comparisons.

## 3 Results and Discussion

### 3.1 Conventional Radial Distribution Function and Coordination Number Analysis

The solvation structure of the 0.1 M NaPF_6_/EC electrolyte at 233.15, 273.15, and 333.15 K was first characterized using conventional radial distribution function (RDF) and coordination number (CN) analyses (Figure 3). The RDF profiles (Figure 3a,b) reveal that the first coordination peak of Na^+^ with the carbonyl oxygen (O_c_) of EC remains centered at 2.68 Å across all temperatures. The intensity of this peak is consistently higher than that of the corresponding oxygen (O_e_) peak, confirming that EC molecules predominantly coordinate with Na^+^ via the more electronegative carbonyl oxygen atom.

**FIGURE 3:**
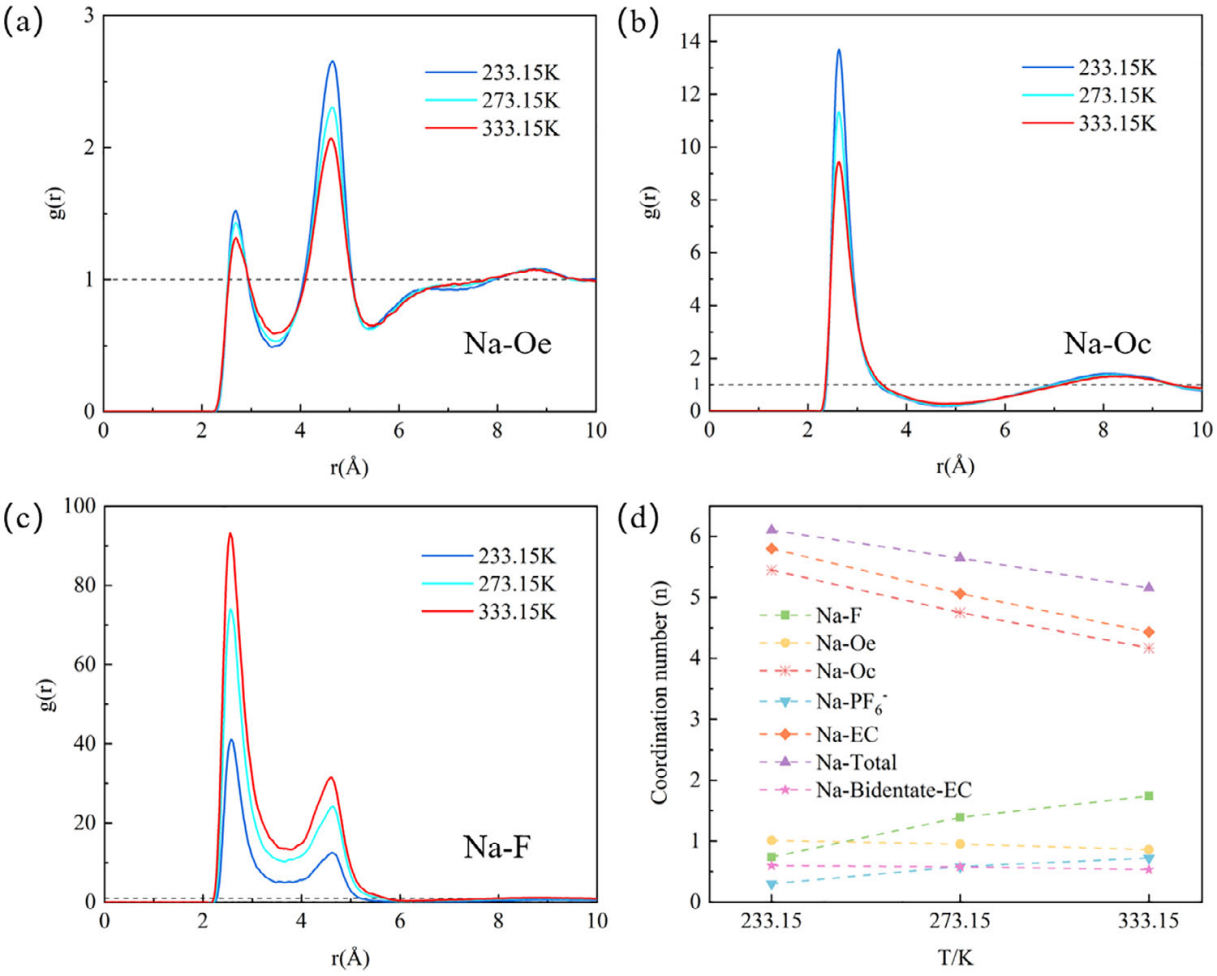
Solvation structure analysis of Na^+^ in the 0.1 M NaPF_6_/EC electrolyte at 233.15, 273.15, and 333.15 K. (a–c) Radial distribution functions (RDFs) of Na^+^ with the ether oxygen (Oe) and carbonyl oxygen (Oc) of EC, and the fluorine atom (F) of PF_6_^−^. (d) Temperature evolution of atomic and molecular coordination numbers (CN). “Na‐Bidentate‐EC” denotes the bidentate coordination mode where both O_e_ and O_c_ of a single EC molecule participate simultaneously.

As the temperature increases, the heights of the first peaks for both Na^+^‐Oc and Na^+^‐Oe gradually decrease. This trend indicates that thermal motion weakens the average coordination bond strength between Na^+^ and EC solvent molecules. Conversely, the height of the first Na^+^‐F peak increases monotonically with rising temperature (Figure 3c), implying that more PF_6_^−^ anions penetrate the first solvation shell of Na^+^ at elevated temperatures. We calculated the average CN to quantify these changes in molecular coordination (Figure 3d). The results show that the average CN of Na^+^ remains relatively constant at five to six across the entire temperature range. However, the CN contributed by EC molecules decreases from 5.8 at 233.15 K to 4.4 at 333.15 K, while the CN contributed by PF_6_^−^ anions increases correspondingly from 0.3 to 0.7. This temperature‐dependent trend is in excellent quantitative agreement with the experimental Raman spectroscopy data reported by Forero et al. [44], validating the reliability of our MD simulations. To further corroborate the statistical reliability of these average CN values, the frame‐by‐frame evolution of the Na^+^—EC, Na^+^—bidentate EC, and Na^+^—PF_6_^−^ coordination numbers throughout the NVT production runs is presented in Figures S4–S12, confirming that the systems are well‐equilibrated and that the coordination environments are stable over the 20 ns simulation time.

A closer examination of the RDF curves, however, shows that the Na^+^ first solvation shell is not a single, well‐defined radial layer. The first peak of the Na^+^–Oc pair correlation exhibits pronounced asymmetric broadening with an extended tail into the 3.5–5.0 Å region (Figure 3b). In this same radial interval, the Na^+^–Oe and Na^+^–F correlations develop secondary maxima; for Na^+^–Oe this outer maximum is more pronounced than its inner one, whereas for Na^+^–F the first‐shell maximum remains dominant. This radial ordering indicates that different ligand atoms occupy distinct distance ranges within the first‐shell region: carbonyl oxygens dominate the inner coordination sites, whereas ether oxygens—constrained by the EC ring geometry—populate predominantly outer distances, and PF_6_^−^ fluorines contribute in the inner shell and, more weakly, in the boundary region.

These observations delineate the intrinsic limitation of conventional RDF/CN analyses. Because spherical averaging collapses all directional information onto the distance axis, it cannot resolve which solvation motif a given snapshot belongs to: configurations with the same total CN but drastically different ligand arrangements—notably coexisting SSIP and CIP—are mapped onto indistinguishable averages. It therefore cannot provide the thermodynamic populations of these conformations, nor the per‐snapshot arrangement of ligands. This loss of motif‐level information is precisely what the frame‐by‐frame SDV—DBSCAN classification in Section 3.2 restores, and it underpins the energy–population–transport chain developed in Sections 3.3.

### 3.2 Solvation Conformation Identification and Temperature Evolution via SDV‐DBSCAN

To address these limitations inherent in conventional RDF/CN analyses, we employed the SDV‐DBSCAN framework developed in this work to perform unsupervised classification of the instantaneous solvation conformations of each Na^+^ ion across the entire trajectory. Following systematic parameter optimization as described in Section 2.3, the clustering parameters were set to ε = 0.8–1.0 Å and MinPts = 100. This parameterization successfully identified two primary statistically stable solvation conformations, with a noise fraction of less than 1% across all temperatures.

The two identified conformations correspond to the well‐known SSIP and CIP configurations. The SSIP conformation (Figure 4a) demonstrates that the first solvation shell is exclusively occupied by oxygen atoms from EC molecules, with all PF_6_^−^ anions located outside the shell and separated from Na^+^ by at least one layer of solvent molecules. Whereas the CIP conformation (Figure 4b) shows that the first solvation shell contains both oxygen atoms from EC molecules and at least one fluorine atom from a PF_6_^−^ anion, indicating direct electrostatic interaction between the cation and anion. We collected the precise statistical proportions of these two conformations at the three investigated temperatures (Figure 4c). As temperature increases from 233.15 to 333.15 K, the proportion of the SSIP conformation decreases significantly from 79.9% to 38.3%, while the proportion of the CIP conformation increases synchronously from 20.1% to 61.7%. This evolutionary trend is in perfect qualitative and quantitative agreement with the RDF/CN analysis in Section 3.1, which showed an increase in PF_6_^−^ coordination number with temperature. These absolute per‐frame populations are the distinguishing output of the present partition. Whereas descriptor‐level and cutoff‐based schemes are designed to characterize the landscape of local environments or to census coordination species and aggregates [22, 23], the per‐frame SDV–DBSCAN partition returns the population of each conformational basin on every frame and resolves high‐energy transition states as explicit noise—the quantities that directly drive the vertical‐desolvation‐energy and transport analyses in Sections 3.3 and 3.4.

**FIGURE 4:**
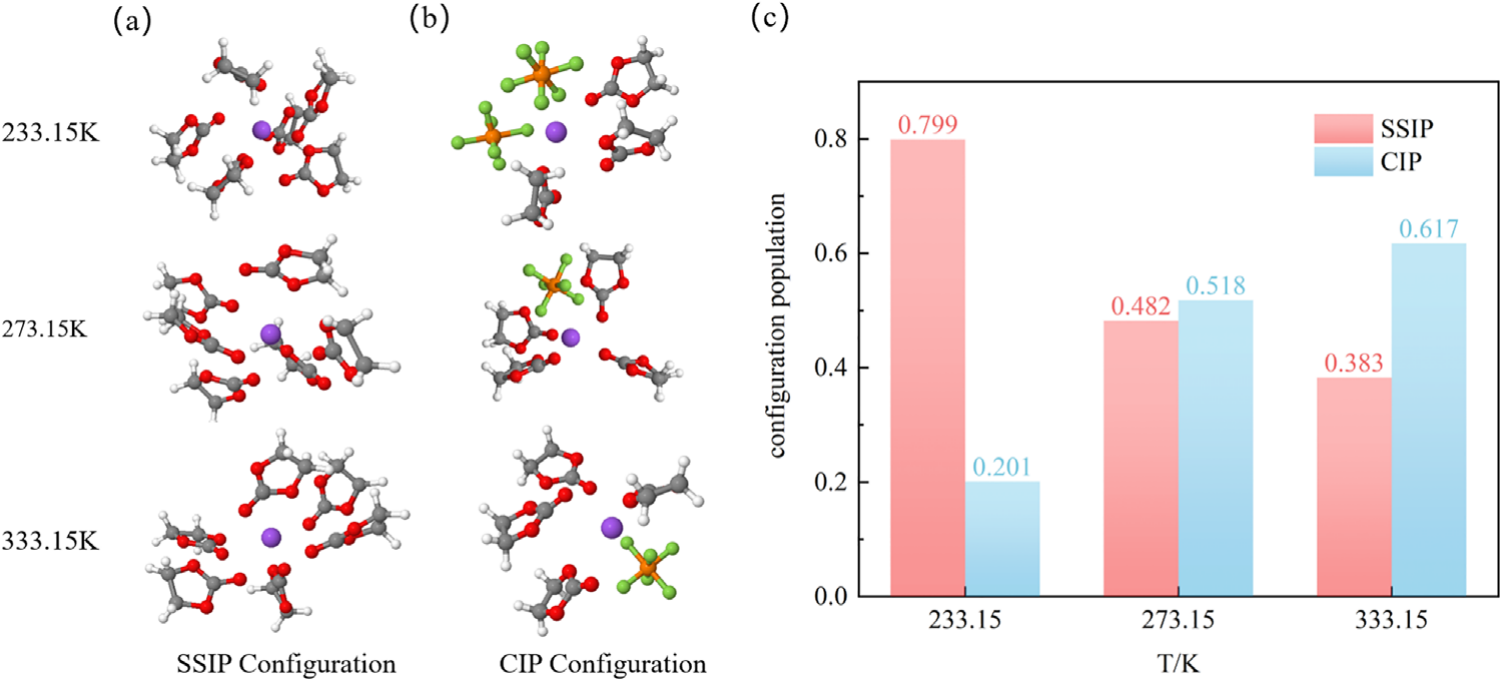
Na^+^ solvation conformations identified via the SDV‐DBSCAN method. (a, b) Representative snapshots of the solvent‐separated ion pair (SSIP) and contact ion pair (CIP) conformations. (c) Statistical temperature dependence of the SSIP and CIP conformation proportions.

More importantly, the SDV‐DBSCAN method provides, for the first time, an absolute quantitative measurement of the SSIP and CIP conformational populations. This capability reveals the rich conformational diversity that is completely masked by the averaging effect of conventional methods. The ability to distinguish and quantify these distinct conformations is critical for understanding the temperature‐dependent performance of SIB electrolytes, as SSIP and CIP exhibit vastly different desolvation kinetics and interfacial reactivity.

The frame‑resolved SSIP/CIP populations obtained here carry a direct thermodynamic meaning that also defines the scope of the present model. The SSIP‐to‐CIP equilibrium is governed by activities, a = γc: for NaPF_6_ in EC the ion‑pair dissociation energy (0.34 kcal·mol^−1^) lies below the thermal energy, so the mean ionic activity coefficient approaches unity [45], and the population ratio at 0.1 M therefore reads out the intrinsic equilibrium constant K(T) itself. The competition we resolve — progressive weakening of Na^+^–EC coordination with relatively strengthened cation–anion association upon heating — is a property of K(T) and of its enthalpic temperature dependence, both intrinsic to the Na^+^/EC/PF_6_^−^ couple and independent of the nominal concentration. At practically relevant compositions, γ± and the thermodynamic factor depart from unity as ion–ion correlations grow, shifting the absolute populations toward contact and higher‑order species while leaving K(T) and its temperature dependence intact; the trend established here accordingly transfers across concentrations, with only the absolute populations requiring a concentration‑specific calibration through γ±. This concentration dependence is borne out experimentally, where 1 M NaPF_6_ in carbonate mixtures shows a comparably strong temperature dependence of ionic transport over a wide temperature window [27]. Because the per‑frame density partition assigns each configuration to a conformational basin by density connectivity, higher‑order aggregates that grow at elevated concentration enter naturally as additional resolved basins, and coupling the physically anchored SDV representation to machine‑learning potentials will carry the same population–energy–transport chain into that regime.

### 3.3 Thermodynamic Mechanisms and Kinetic Impacts of Temperature‐Driven Conformational Transitions

To elucidate the molecular‐level mechanism underlying the temperature‐induced conformational evolution, we performed high‐precision DFT vertical single‐point energy calculations on the representative conformations extracted using the centroid minimum distance method (Section 2.4). The calculations demonstrate that across all tested temperatures, the single‐point energy of the CIP conformation is significantly lower than that of the SSIP conformation (Figure 5a). This indicates that at the local electronic structure level, direct contact between Na^+^ and PF_6_^−^ places the CIP conformation in a deeper potential energy well, conferring greater intrinsic electronic stability.

**FIGURE 5:**
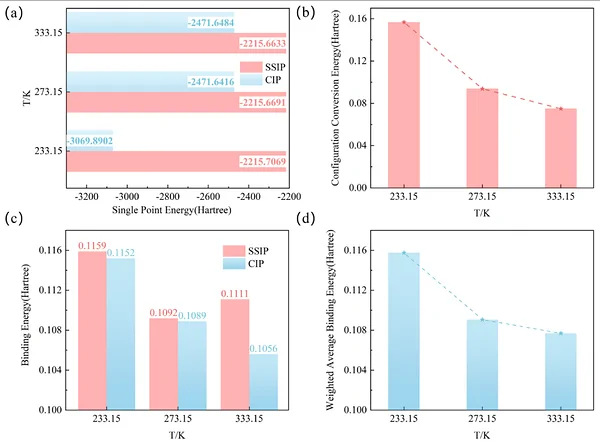
Desolvation energetics derived from DFT vertical single‐point energy calculations. (a) Relative vertical energies of SSIP and CIP conformations. (b) Thermodynamic energy differences (energy penalties) for the structural transition from SSIP to CIP. (c) Vertical binding energy (*E*_bind_) between Na^+^ and its first solvation shell in isolated SSIP and CIP conformations. (d) System‐weighted average binding energy calculated based on MD conformation proportions.

Further analysis reveals that the structural transition from SSIP to CIP proceeds via distinct thermodynamic pathways as a function of the temperature. At low temperatures (233.15 K), the formation of CIP‐2 (containing two PF_6_^−^ anions in the first solvation shell) requires a two‐step process: initial conversion of SSIP to CIP‐1 (containing a single PF_6_^−^ anion), followed by the insertion of a second anion:

$${\mathrm{SSIP}+\mathrm{PF}}_{6}^{-}\;\to \;\mathrm{CIP}-1\;(\Delta E_{1})$$

$${\mathrm{CIP}-1+\mathrm{PF}}_{6}^{-}\;\to \;\mathrm{CIP}-2\;(\Delta E_{2})$$

As shown in Figure 5b, the total energy penalty (Δ*E*_1_ + Δ*E*_2_) for SSIP‐to‐CIP‐2 reaches 98.3 kcal/mol (0.157 hartree) at 233.15 K. At higher temperatures (273.15–333.15 K), however, the system predominantly forms CIP‐1, and the energy difference for SSIP‐to‐CIP‐1 decreases to 47.0 kcal/mol (0.075 hartree) at 333.15 K. It should be noted that these ΔE_conv_ values are thermodynamic energy differences between stable conformational basins, calculated from vertical DFT single‑point energies rather than kinetic activation free energies obtained from transition‑state searches or free‑energy sampling. The physical rationale for using this thermodynamic penalty as a semi‑quantitative metric for conversion propensity rests on the Hammond postulate for solution‑phase ion transfer: the transition state for solvent shell rearrangement and anion penetration closely resembles the CIP product state (a late barrier), so the thermodynamic energy difference constitutes the dominant contribution to the true activation barrier. A detailed demonstration is provided in Section S5. This approach of quantifying SSIP–CIP conformational preferences and conversion tendencies via relative DFT energies have precedent in computational battery electrolyte research [46].

This substantial reduction in the energy penalty for anion insertion, combined with the increased thermal kinetic energy of the system at high temperatures, provides a clear microscopic explanation for the significant increase in CIP proportion with rising temperature. It also accounts for the low population of CIP‐2 at low temperatures, where the large energy penalty disfavors the second anion insertion step. The raw DFT single‐point energies for all extracted SSIP and CIP configurations, along with the isolated EC and PF_6_^−^ reference energies used to calculate these conformational transition energies, are summarized in Table S2.

To quantify the energy threshold for interfacial desolvation—the rate‑determining step for Na^+^ transport across the electrode‑electrolyte interface and the primary bottleneck for low‑temperature battery performance—we calculated the binding energy (E_bind_) between Na^+^ and its first solvation shell, defined as:

$$E_{\mathrm{bind}}=\left(E_{\mathrm{Na}}+E_{\mathrm{shell}}\right)-E_{\mathrm{complex}}$$

where *E*_complex_ is the total energy of the intact solvation cluster, and *E*_Na_ and *E*_shell_ are the energies of the isolated Na^+^ cation and the remaining solvent shell after removal of Na^+^, respectively. This binding energy metric is widely established as a reliable descriptor of interfacial desolvation kinetics [47, 48]: a higher *E*_bind_ value indicates stronger attractive interaction between Na^+^ and its surrounding solvation shell, corresponding to a higher kinetic barrier that must be overcome for desolvation to proceed at the electrode surface.

Across all temperatures, the binding energy of the CIP conformation is consistently lower than that of the SSIP conformation (Figure 5c). The underlying mechanism is that the strong electrostatic interaction between Na^+^ and PF_6_^−^ in the CIP conformation partially replaces the weaker Na^+^‐EC coordination bonds. Additionally, the steric bulk of the PF_6_^−^ anion disrupts the close packing of solvent molecules around Na^+^, resulting in a loose, easily disrupted solvation shell.

To establish a quantitative link between microscopic conformational populations and macroscopic kinetic properties, we calculated the weighted average binding energy of the system using the MD‐derived conformational proportions. The weighted average binding energy decreases from 72.6 kcal/mol (0.116 Hartree) at 233.15 K to 67.6 kcal/mol (0.108 Hartree) at 333.15 K (Figure 5d). The detailed DFT‐calculated binding energies for individual conformations, the corresponding conformational weights, and the final weighted average binding energies are compiled in Table S3. This downward trend in the average desolvation energy barrier predicted by DFT is highly consistent with the macroscopic kinetic results obtained from MD simulations.

The mean square displacement (MSD) curves (Figure 6a–c) and the quantitative ionic mobility analysis (Figure 6d) demonstrate that the Na^+^ diffusion rate increases dramatically with temperature, with ionic mobility improving by more than an order of magnitude between 233.15 and 333.15 K (Table S3). Taken together, these results establish a complete and self‐consistent mechanistic closed loop: elevated temperatures lower the SSIP‐CIP energy penalty, promoting CIP formation, which in turn reduces the average desolvation barrier and ultimately enhances macroscopic ionic mobility. The comprehensive methodology for calculating the Na^+^ diffusion coefficients and ionic mobilities, including trajectory unwrapping, FFT （Fast Fourier Transform）‐based MSD calculation, and the application of the Einstein and Nernst‐Einstein relations, is detailed in Section S6.

**FIGURE 6:**
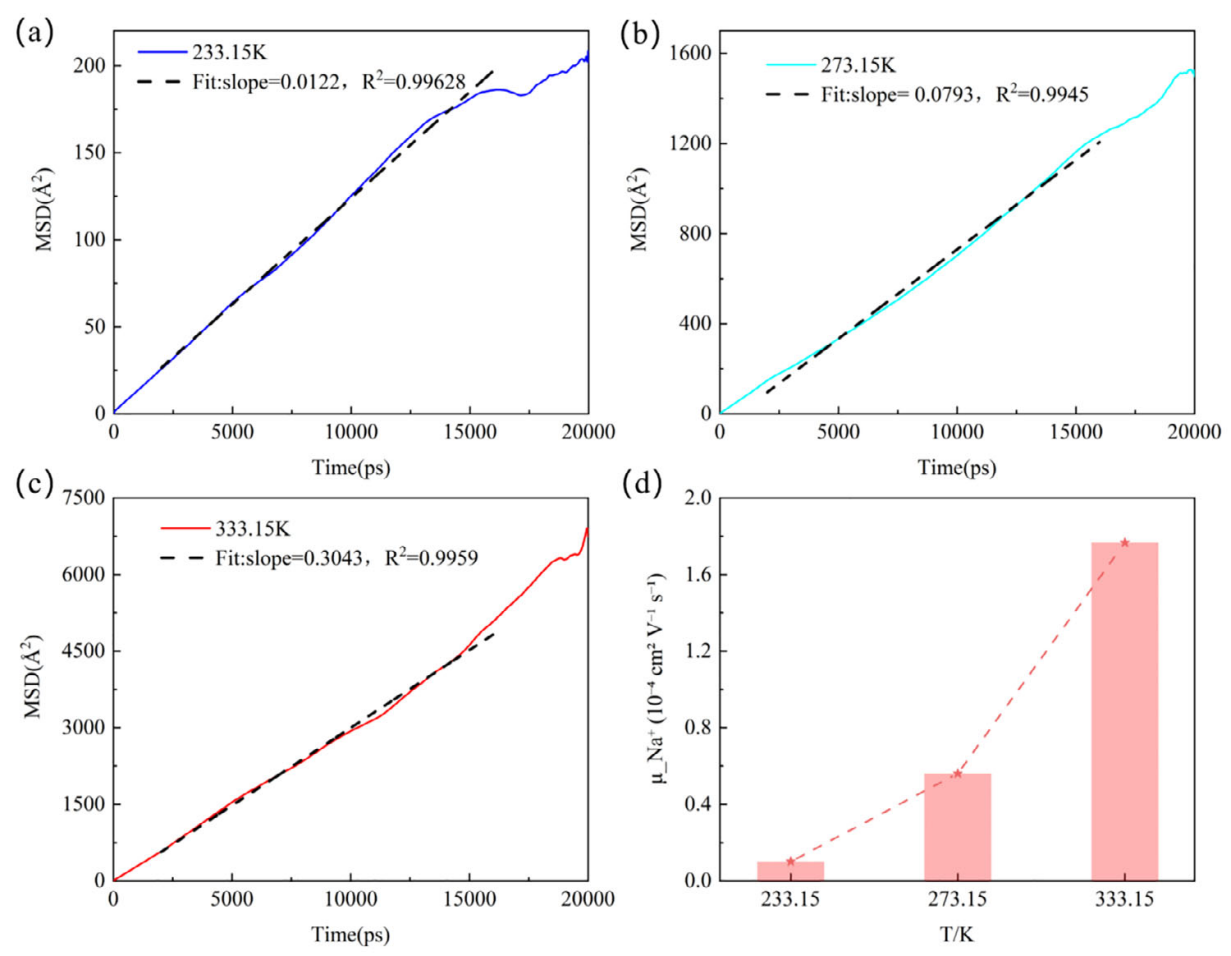
Macroscopic transport kinetics of Na^+^ derived from MD simulations. (a–c) Mean square displacement (MSD) of Na^+^ over time at 233.15, 273.15, and 333.15 K. (d) Temperature evolution of Na^+^ ionic mobility.

This mechanism not only explains the temperature‐dependent transport properties of NaPF_6_/EC electrolytes but also provides a clear molecular design principle for wide‐temperature SIB electrolytes. Strategies that promote the formation of CIP conformations at low temperatures will reduce the desolvation energy barrier and improve low‐temperature performance. Conversely, at elevated temperatures, it is essential to preserve an adequate fraction of SSIP conformations to avoid excessive ion‐pair aggregation and over‐weakening of the solvation structure, thereby ensuring robust and sustained Na^+^ transport kinetics.

From solvation structure to descriptor‐based electrolyte design. The quantities established above form a compact and physically transparent descriptor set for Na^+^ electrolytes: the SSIP and CIP populations with their temperature dependence from the frame‐by‐frame SDV–DBSCAN classification, the energy penalty ΔE_conv_ of Na^+^ desolvation from DFT, and the Na^+^ mobility derived from mean‐squared‐displacement analysis. All three are obtained for a given composition within a single bulk simulation workflow and therefore transfer to other solvent, salt, and additive combinations without re‐parameterization. More importantly, they are not a list of unrelated numbers but the three links of the chain identified above: the motif populations fix how many ion pairs must overcome ΔE_conv_ before contributing to transport, and ΔE_conv_ governs the mobility of the carriers that do. They can thus serve as physically interpretable features rather than black‐box inputs. A descriptor‐based design cycle follows directly—enumerate candidate solvent/salt/additive combinations, evaluate this descriptor set for each, regress a surrogate model linking them to the transport performance of interest, and invert the surrogate by Bayesian optimization or generative molecular design to nominate candidates for experimental screening. Recent AI‐ and data‐driven studies of battery electrolytes and interphases have established this microscopic‐to‐macroscopic mapping as a working paradigm, and have identified the shortage of physically meaningful and transferable descriptors as the principal obstacle to its wider use [49]. The unsupervised partitioning of a descriptor space exploited in those studies [50] belongs to the same class of methods as our SDV–DBSCAN classification, and the experimentally validated screening reported there indicates how the cycle above is closed in practice. Within the present work, the analysis is focused on bulk solvation, where the transport bottleneck of the systems studied here originates. Extending the descriptor set toward interfacial quantities is a natural continuation: the bulk solvation structure and binding strength characterized here set the baseline energy cost for desolvation at the electrode–electrolyte interface, and the SSIP/CIP conformational ratio further modulates how readily that desolvation proceeds.

## 4 Conclusions

In this work, we developed an automated conformational clustering framework integrating sorted distance vector (SDV) feature engineering and DBSCAN density‐based clustering to investigate the temperature‐dependent solvation evolution of Na^+^ in a 0.1 M NaPF_6_/EC electrolyte across 233.15–333.15 K. This approach overcomes the inherent spherical‐averaging limitation of conventional RDF analysis, enabling quantitative resolution of the microscopic conformational inhomogeneity that underpins electrolyte transport and desolvation kinetics. The main findings and implications are summarized as follows.

First, the temperature‐dependent population shift between SSIP and CIP conformations, previously obscured by ensemble averaging, is quantitatively resolved. The SDV–DBSCAN framework identifies two distinct stable solvation motifs: solvent‐separated ion pairs, where the first coordination shell is exclusively occupied by EC solvent molecules, and contact ion pairs, where PF_6_^−^ anions enter the inner shell and directly coordinate with Na^+^. As temperature rises from 233.15 to 333.15 K, the SSIP fraction decreases from 79.9% to 38.3%, while the CIP fraction increases correspondingly from 20.1% to 61.7%. This frame‐resolved quantification of conformational populations provides a microscopic basis for understanding the temperature‐dependent performance of sodium‐ion electrolytes.

Second, a complete mechanistic closed loop linking microscopic conformational transitions to macroscopic ionic mobility is established. DFT vertical single‐point energy calculations show that elevated temperatures drastically reduce the thermodynamic SSIP‐to‐CIP energy penalty ΔE_conv_ — from 98.3 kcal/mol at 233.15 K to 47.0 kcal/mol at 333.15 K — shifting the conformational equilibrium toward the more thermodynamically stable CIP state. Importantly, CIP conformations exhibit a lower Na^+^–shell binding energy E_bind_ than SSIP conformations. Weighted by the MD‐derived conformational populations, the system‐averaged desolvation energy barrier decreases from 72.6 to 67.6 kcal/mol over the investigated temperature range, which directly accounts for the order‐of‐magnitude enhancement in Na^+^ ionic mobility observed in MD simulations. In short, higher temperatures lower the SSIP‐to‐CIP conversion penalty, increase the CIP population, and reduce the average desolvation barrier, collectively enhancing macroscopic ion transport.

Third, the temperature‐dependent trend is intrinsic to the Na^+^/EC/PF_6_^−^ couple and transferable to practical electrolyte compositions. The SSIP⇌CIP equilibrium is governed by the intrinsic equilibrium constant K(T), whose enthalpic temperature dependence is set by the cation–solvent and cation–anion interactions, independent of nominal salt concentration [45]. At practical higher concentrations, the mean activity coefficient γ_±_ calibrates the absolute populations toward more contact species, but the temperature dependence of K(T) and the underlying mechanistic trend remain intact. This is consistent with experimental observations of comparable temperature dependence of ionic transport in concentrated carbonate‐based sodium electrolytes.

The mechanistic picture established here provides explicit molecular engineering guidelines for designing wide‐temperature sodium‐ion electrolytes. Electrolyte formulations that promote CIP formation at low temperatures can effectively reduce the desolvation energy barrier and improve low‐temperature performance; conversely, at elevated temperatures, preserving an adequate SSIP fraction prevents excessive ion aggregation and over‐weakening of the solvation structure, ensuring robust and sustained Na^+^ transport under high‐temperature conditions.

Finally, the SSIP/CIP populations, the conformational energy penalty ΔEconv, and the Na^+^ mobility derived in this work form a compact, physically interpretable descriptor set, all obtained from a single bulk simulation workflow. Coupling these physically anchored descriptors with device‐level performance metrics via surrogate models and Bayesian or generative optimization represents a promising pathway toward data‐driven and AI‐assisted electrolyte design [49, 50]. Ultimately, this mechanism‐driven analytical framework provides a quantitative tool for the rational inverse design of advanced sodium‐ion electrolytes operating across extreme temperature ranges.

## Author Contributions

**Long Huang**: writing – original draft, conceptualization, methodology, data curation, formal analysis, visualization. **Kun Gao**: investigation, validation. **Yiyao Han**: validation, formal analysis. **Jing Yang**: funding acquisition, writing – review and editing, supervision, resources, project administration, data curation, software.

## Conflicts of Interest

The authors declare no conflicts of interest.

## Supporting information




**Supporting File**: advs78718‐sup‐0001‐SuppMat.docx.

## Data Availability

The data supporting the findings of this study, including all simulation input files, key analysis scripts, and summarized numerical data, are openly available in the Zenodo repository at https://doi.org/10.5281/zenodo.21651279. The large raw trajectory files are not publicly available due to their excessive size, and can be obtained from the corresponding author upon reasonable request.
