# Supplementary material for: Automated Conformational Clustering Reveals Desolvation Mechanisms in a Wide‐Temperature‐Range Sodium‐Ion Electrolytes
Source: Adv Sci (Weinh). 2026 Nov 9:e78718. Online ahead of print. doi: 10.1002/advs.78718 (PMC13748889; doi:10.1002/advs.78718)
Supplement: Supplementary file 1 [file ADVS-9999-e78718-s001.docx]

Automated conformational clustering reveals desolvation mechanisms in wide-temperature sodium-ion electrolytes

Long Huang , Kun Gao, Yiyao Han, Jing Yang*

Jing Yang. Corresponding Author
School of Chemical Engineering and Technology, Sun Yat-sen University, Zhuhai, 519082, Guangdong P. R. China

E-mail: [yangjing25@mail.sysu.edu.cn](mailto:yangjing25@mail.sysu.edu.cn)

Supporting Information Content

[Table S1. Summary of Key Parameters for MD Simulation	3](#_Toc242822935)

[Figure S1. Density convergence and equilibration in NPT ensemble at 233.15 K. The blue solid line shows the cumulative mean density, the light blue shaded region represents the 95% confidence interval, and the red dashed line indicates the equilibrated density (1.3628 g/cm³). Equilibration begins at approximately 3.5 ns, as indicated by the vertical dashed line. All simulations were performed under NPT conditions (1 atm, 233.15 K).	5](#_Toc242822936)

[Figure S2. Density convergence and equilibration in NPT ensemble at 273.15 K. The blue solid line shows the cumulative mean density, the light blue shaded region represents the 95% confidence interval, and the red dashed line indicates the equilibrated density (1.3197 g/cm³). Equilibration begins at approximately 3.5 ns, as indicated by the vertical dashed line. All simulations were performed under NPT conditions (1 atm, 273.15 K).	6](#_Toc242822937)

[Figure S3. Density convergence and equilibration in NPT ensemble at 333.15 K. The blue solid line shows the cumulative mean density, the light blue shaded region represents the 95% confidence interval, and the red dashed line indicates the equilibrated density (1.2546 g/cm³). Equilibration begins at approximately 3.5 ns, as indicated by the vertical dashed line. All simulations were performed under NPT conditions (1 atm, 333.15 K).	6](#_Toc242822938)

[Figure S4. Variation of Na⁺-bidentate EC coordination number as a function of simulation frame at 233.15 K. Green vertical lines represent instantaneous coordination values for each frame, and the black dashed line indicates the equilibrated average coordination number (0.5977). Total simulation time: 5 ns (8000 frames).	7](#_Toc242822939)

[Figure S5. Variation of Na⁺-bidentate EC coordination number as a function of simulation frame at 273.15 K. Green vertical lines represent instantaneous coordination values for each frame, and the black dashed line indicates the equilibrated average coordination number (0.5863). Total simulation time: 5 ns (8000 frames).	7](#_Toc242822940)

[Figure S6. Variation of Na⁺-bidentate EC coordination number as a function of simulation frame at 333.15 K. Green vertical lines represent instantaneous coordination values for each frame, and the black dashed line indicates the equilibrated average coordination number (0.5286). Total simulation time: 5 ns (8000 frames).	8](#_Toc242822941)

[Figure S7. Variation of Na⁺-PF6- coordination number as a function of simulation frame at 233.15 K. Red vertical lines represent instantaneous coordination values for each frame, and the black dashed line indicates the equilibrated average coordination number (0.2999). Total simulation time: 5 ns (8000 frames).	8](#_Toc242822942)

[Figure S8. Variation of Na⁺-PF6- coordination number as a function of simulation frame at 273.15 K. Red vertical lines represent instantaneous coordination values for each frame, and the black dashed line indicates the equilibrated average coordination number (0.5440). Total simulation time: 5 ns (8000 frames).	9](#_Toc242822943)

[Figure S9. Variation of Na⁺-PF6- coordination number as a function of simulation frame at 333.15 K. Red vertical lines represent instantaneous coordination values for each frame, and the black dashed line indicates the equilibrated average coordination number (0.7509). Total simulation time: 5 ns (8000 frames).	9](#_Toc242822944)

[Figure S10. Variation of Na⁺-EC coordination number as a function of simulation frame at 233.15 K. Blue vertical lines represent instantaneous coordination values for each frame, and the black dashed line indicates the equilibrated average coordination number (5.8011). Total simulation time: 5 ns (8000 frames).	10](#_Toc242822945)

[Figure S11. Variation of Na⁺-EC coordination number as a function of simulation frame at 273.15 K. Blue vertical lines represent instantaneous coordination values for each frame, and the black dashed line indicates the equilibrated average coordination number (5.1121). Total simulation time: 5 ns (8000 frames).	10](#_Toc242822946)

[Figure S12. Variation of Na⁺-EC coordination number as a function of simulation frame at 333.15 K. Blue vertical lines represent instantaneous coordination values for each frame, and the black dashed line indicates the equilibrated average coordination number (4.3900). Total simulation time: 5 ns (8000 frames).	11](#_Toc242822947)

[Table S2. Table S3. Summary of DFT single-point energies for SSIP and CIP ion-pair configurations extracted from NPT-equilibrated MD trajectories at 233.15, 273.15, and 333.15 K, along with isolated EC and PF₆⁻ reference energies used for conformational transition energy calculations.	16](#_Toc242822948)

[Table S3. DFT-calculated binding energies of Na⁺ in SSIP and CIP configurations, weighted average binding energies, and corresponding Na⁺ ionic mobilities from MD simulations.	16](#_Toc242822949)

# 1. Molecular Dynamics (MD) Simulations

## 1.1 Force Field Parameter Details

All-atom molecular dynamics simulations were performed using LAMMPS (version 2 Aug 2023)^1^ on the Sugon supercomputing platform (Hefei Advanced Computing Center, with multi-core CPU parallelization). The interactions within the 0.1 M NaPF_6_/EC electrolyte system were described by the OPLS-AA force field, with the initial topologies and force field parameters constructed via Moltemplate^2^. The parameter sources for each component are detailed as follows:

Ethylene Carbonate (EC): The topology and force field parameters (Lennard-Jones parameters and partial charges) were automatically generated through the LigParGen web server^3, 4^, with partial atomic charges assigned using the 1.14CM1A-LBCC scheme^5^.

Sodium Ion (Na^+^): The Lennard-Jones parameters were adopted from the OPLS-AA 2024 force field parameter set developed by the Jorgensen group^6^, invoked via the oplsaa2024.lt library in Moltemplate.

Hexafluorophosphate Anion (PF_6_^-^): The force field parameters were taken from the recalibrated anion parameter set for the OPLS force field developed by Doherty et al^7^.

Mixing Rules: The geometric mixing rule ( $\sigma_{ij}=\sqrt{\sigma_{i}\sigma_{j}}, \varepsilon_{ij}=\sqrt{\varepsilon_{i}\varepsilon_{j}}$ ) was applied to the Lennard-Jones parameters for unlike atom pairs.

Table S1. Summary of Key Parameters for MD Simulation

| Parameters | Numerical / Method |
| --- | --- |
| Simulation Software | LAMMPS (2 Aug 2023) |
| Topology Construction | Moltemplate |
| Simulated Ensemble | NPT（Equilibration）+ NVT（Production Run） |
| PPPM | 1.0×10 −5 |
| Time Step | 0.5 fs |
| Simulation Duration | NPT 5 ns + NVT 20 ns |
| Track Save Interval | 2.5 ps |
| Simulated Temperature | 233.15 K、273.15 K、333.15 K |
| System Composition | 1500 EC + 10 NaPF₆（~0.1 mol/L，Box side length ~55 Å） |

## 1.2 Force-Field Validation and Low-Temperature Phase State

The assembled OPLS-AA force field was checked against available physical properties rather than merely adopted. The NPT equilibrium densities are 1.3628, 1.3197 and 1.2546 g cm^-3^ at 233.15, 273.15 and 333.15 K; these are within about 3% of the experimental density of EC (about 1.32 g cm^-3^ near its melting point and 1.3383 g cm^-3^ for supercooled EC at 25 °C)^8, 9^. The simulated first-shell coordination numbers and their temperature evolution reproduce the experimental variable-temperature Raman trends (main text Fig. 3d), supporting the structural and mechanistic conclusions. The equilibrium freezing transition is not a variable in the present analysis, as our conclusions rest on relative temperature‑dependent trends within the liquid state; quantitative phase coexistence calculations are not required for the current scope and can be added in future extensions of the framework.”

Accordingly, the 233.15 and 273.15 K runs are interpreted as metastable supercooled liquids. EC supercools readily^9^, and in a roughly 55 Å box over 20 ns homogeneous nucleation is not observed; the density and MSD traces remain continuous and liquid-like. As an ideal estimate, dissolving 0.1 M NaPF_6_ depresses the EC freezing point by only ΔT_f_ ≈ i K_f_ m ≈ 2 × 5.39 × 0.076 ≈ 0.8 °C, so the low-temperature liquid state is kinetically stabilized through supercooling rather than thermodynamically stabilized. Practical formulations blend EC with low-melting co-solvents to extend the liquid range^8^.

## 1.3 Representative MD Results

(1) Validation of density equilibration in the NPT ensemble

Figure S1. Density convergence and equilibration in NPT ensemble at 233.15 K. The blue solid line shows the cumulative mean density, the light blue shaded region represents the 95% confidence interval, and the red dashed line indicates the equilibrated density (1.3628 g/cm³). Equilibration begins at approximately 3.5 ns, as indicated by the vertical dashed line. All simulations were performed under NPT conditions (1 atm, 233.15 K).

Figure S2. Density convergence and equilibration in NPT ensemble at 273.15 K. The blue solid line shows the cumulative mean density, the light blue shaded region represents the 95% confidence interval, and the red dashed line indicates the equilibrated density (1.3197 g/cm³). Equilibration begins at approximately 3.5 ns, as indicated by the vertical dashed line. All simulations were performed under NPT conditions (1 atm, 273.15 K).

Figure S3. Density convergence and equilibration in NPT ensemble at 333.15 K. The blue solid line shows the cumulative mean density, the light blue shaded region represents the 95% confidence interval, and the red dashed line indicates the equilibrated density (1.2546 g/cm³). Equilibration begins at approximately 3.5 ns, as indicated by the vertical dashed line. All simulations were performed under NPT conditions (1 atm, 333.15 K).

（2）Frame-by-frame coordination number analysis in the NVT ensemble

Figure S4. Variation of Na⁺-bidentate EC coordination number as a function of simulation frame at 233.15 K. Green vertical lines represent instantaneous coordination values for each frame, and the black dashed line indicates the equilibrated average coordination number (0.5977). Total simulation time: 5 ns (8000 frames).

Figure S5. Variation of Na⁺-bidentate EC coordination number as a function of simulation frame at 273.15 K. Green vertical lines represent instantaneous coordination values for each frame, and the black dashed line indicates the equilibrated average coordination number (0.5863). Total simulation time: 5 ns (8000 frames).

Figure S6. Variation of Na⁺-bidentate EC coordination number as a function of simulation frame at 333.15 K. Green vertical lines represent instantaneous coordination values for each frame, and the black dashed line indicates the equilibrated average coordination number (0.5286). Total simulation time: 5 ns (8000 frames).

Figure S7. Variation of Na⁺-PF6- coordination number as a function of simulation frame at 233.15 K. Red vertical lines represent instantaneous coordination values for each frame, and the black dashed line indicates the equilibrated average coordination number (0.2999). Total simulation time: 5 ns (8000 frames).

Figure S8. Variation of Na⁺-PF6- coordination number as a function of simulation frame at 273.15 K. Red vertical lines represent instantaneous coordination values for each frame, and the black dashed line indicates the equilibrated average coordination number (0.5440). Total simulation time: 5 ns (8000 frames).

Figure S9. Variation of Na⁺-PF6- coordination number as a function of simulation frame at 333.15 K. Red vertical lines represent instantaneous coordination values for each frame, and the black dashed line indicates the equilibrated average coordination number (0.7509). Total simulation time: 5 ns (8000 frames).

Figure S10. Variation of Na⁺-EC coordination number as a function of simulation frame at 233.15 K. Blue vertical lines represent instantaneous coordination values for each frame, and the black dashed line indicates the equilibrated average coordination number (5.8011). Total simulation time: 5 ns (8000 frames).

Figure S11. Variation of Na⁺-EC coordination number as a function of simulation frame at 273.15 K. Blue vertical lines represent instantaneous coordination values for each frame, and the black dashed line indicates the equilibrated average coordination number (5.1121). Total simulation time: 5 ns (8000 frames).

Figure S12. Variation of Na⁺-EC coordination number as a function of simulation frame at 333.15 K. Blue vertical lines represent instantaneous coordination values for each frame, and the black dashed line indicates the equilibrated average coordination number (4.3900). Total simulation time: 5 ns (8000 frames).

# 2. Mathematical Definition and Invariance of the SDV Descriptor

To overcome the limitation of the spherical averaging inherent in the radial distribution function (RDF), we propose the Sorted Distance Vector (SDV) to characterize the instantaneous solvation shell of Na^+^ (see Section 2.2 in the main text). With the Na^+^ as the origin, the sets of distances to all EC oxygen atoms (Oc, Oe) and PF₆⁻ fluorine atoms (F) are computed, denoted as dO and dF, respectively. After sorting in ascending order, the first 10 elements of dO and the first 6 elements of dF are concatenated to form a 16-dimensional vector:

SDV=[d O_1_,d O_2_,...,d O_10_,d F_1_,d F_2_ ,...,d F_6_]

Invariance: Two invariance properties ensure that each solvation configuration obtains a unique representation in the feature space.

(1) Permutation invariance: The sorting operation renders the output invariant to label exchanges among particles of the same type, i.e., sort(π(D))=sort(D) .

(2) Rotational/translational invariance: The descriptor depends solely on scalar Euclidean distances d=||r_i_-r_Na_||, which are invariant under any rigid-body transformation (R,t) , since ||R(r_i_-r_Na_)|| =||r_i_-r_Na_|| .

(3) Dimensionality Justification: The SDV truncation (10O + 6F) reflects the physical coordination capacity of the Na^+^ first solvation shell and the intrinsic geometry of PF_6_^-^.

For oxygen, CN analysis (main text Fig. 3d) indicates that the first shell contains 4–6EC molecules, corresponding to 8–12 coordinating oxygens (each EC contributes both Oc and Oe). Ten oxygen distances fully encompass the primary shell while retaining marginal boundary sensitivity; a smaller cutoff would truncate legitimate shell oxygens at low temperatures, whereas a larger one would introduce bulk noise devoid of conformational information.

For fluorine, each PF_6_^-^ carries 6 equivalent F atoms. In CIP-1, the 6-F window completely encodes the single anion. In CIP-2, electrostatic repulsion and steric constraints force two PF_6_^-^ anions to coordinate Na^+^ at comparable distances. Upon ascending sorting, the 6 F positions naturally interleave between the two anions (typically ~3 nearest F from each), ensuring the window captures a balanced, symmetry-resolved subset of both rather than biasing toward either. Six F dimensions therefore suffice to discriminate CIP-1 and CIP-2 conformations without expanding to 12, which would merely introduce redundant bulk features in this dilute regime.

# 3. DBSCAN Clustering Algorithm and Parameter Selection

Unsupervised classification of solvation configurations was performed using the DBSCAN algorithm^10^ (outlined in Section 2.3 of the main text).

Algorithm Core: DBSCAN identifies cluster structures based on density connectivity. If the ϵ -neighborhood of a data point p contains at least MinPts points, p is designated as a core point. Density-connected core points and their associated border points form a single cluster, while the remaining points are classified as noise.

MinPts = 100: At each temperature, the dataset comprises 8,000 frames × 10 Na^+^ = 80,000 data points. Setting MinPts = 100 requires that each valid cluster contains at least ~0.125% of the total data points, ensuring that only statistically significant configurational basins are retained.

Selection of ϵ : To capture the majority of configurations, the search radius was incrementally increased starting from ϵ = 0.8 Å until the noise fraction dropped below 1%. This threshold is motivated by physical considerations: in equilibrium MD trajectories, over 99% of frames are expected to reside in stable potential energy basins (SSIP or CIP), with only a very small fraction corresponding to high-energy transition states associated with barrier-crossing events. The final ϵ values ranged from 0.8 to 1.0 Å across different temperatures.

Statistical convergence of the SSIP/CIP populations and coordination numbers. Convergence was verified through multiple complementary approaches. The analysis is based on 80,000 per-ion snapshots at each temperature (8,000 frames × 10 Na⁺), providing sufficient statistical sampling of the configurational ensemble. DBSCAN consistently resolves exactly two dominant stable conformational basins across all temperatures with a noise fraction below 1%, confirming that the overwhelming majority of snapshots occupy well-defined energetic minima. The frame-by-frame running averages of the coordination numbers (Figs. S4–S12) converge rapidly after equilibration and remain flat for the remainder of the production run, with no systematic drift. The population-derived average coordination numbers are also in quantitative agreement with conventional RDF results, providing independent cross-validation of the classification statistics.

# 4. Density Functional Theory (DFT) Computational Setup

All quantum chemical calculations were performed using ORCA 6.0.1^11-13^ (outlined in Section 2.4 of the main text). The extracted representative snapshots were used without geometric relaxation to preserve the authentic thermal fluctuation characteristics. Vertical single-point energy calculations were carried out at the B3LYP-D3(BJ)/6-31G(d,p) level with the TightSCF convergence criterion. The ORCA keyword line is as follows:

! B3LYP D3BJ 6-31G(d,p) TightSCF CPCM

CPCM Solvation Model: To account for the long-range screening effects of the bulk dielectric environment, the Conductor-like Polarizable Continuum Model (CPCM) was employed, uniformly adopting the standard high-dielectric parameters of EC at room temperature ( ε≈90 )^14^ as the background reference. The justification for this approximation is as follows. The core objective of the DFT calculations in this work is to evaluate the short-range quantum chemical interactions of explicitly coordinated molecules within the first solvation shell, while CPCM serves solely to provide a long-range polarization background. In a highly polar carbonate solvent ( ε≫1 ), the influence of the specific background dielectric constant on the local relative energy ordering becomes saturated. Therefore, the uniform use of room-temperature parameters does not alter the qualitative conclusions regarding the relative energy differences between SSIP and CIP, while ensuring consistency across comparisons at different temperatures.

# 5. Thermodynamic Interpretation of the SSIP-CIP Energy Difference

We note that the conformational energy difference ΔEconv reported in the main text is a thermodynamic energy difference between the stable SSIP and CIP potential energy basins, calculated from vertical DFT single‑point energies of representative MD snapshots. It is not a kinetic activation free energy obtained from transition‑state searches, umbrella sampling, or other free‑energy calculation methods. We did not explicitly map the reaction coordinate or sample the transition‑state region.

The physical justification for using this thermodynamic energy penalty as a semi‑quantitative descriptor for conversion propensity is based on the Hammond postulate for solution‑phase ion‑transfer processes. For reactions involving solvent‑shell reorganization and ionic penetration into the first coordination sphere, the transition state structurally resembles the product state more closely than the reactant state (a “late” barrier along the reaction coordinate). This is a well‑established principle for ion‑pairing, ligand exchange, and desolvation processes in liquid electrolyte environments, where solvent reorganization constitutes the dominant energetic contribution to the activation barrier.

In the present system, the SSIP → CIP conversion involves partial stripping of the EC solvation shell around Na⁺ and penetration of a PF₆⁻ anion into the first coordination sphere. The transition state for this process requires breaking of Na⁺–EC coordination bonds and rearrangement of the solvent shell to accommodate the anion, a configuration that is structurally closer to the CIP product state than to the intact SSIP reactant state. Consequently, the thermodynamic energy penalty ΔEconv constitutes the dominant contribution to the true kinetic activation energy for the SSIP‑to‑CIP conversion.

Accordingly, ΔEconv serves as a useful semi‑quantitative thermodynamic metric for comparing the SSIP‑to‑CIP conversion propensity across different temperatures. A larger energy penalty corresponds to a higher kinetic barrier and lower conversion rate, while a reduced penalty at elevated temperatures promotes CIP formation. This approximation is sufficient to establish the mechanistic link between the temperature-dependent conformational shift and the enhanced ionic mobility observed in MD simulations, and this strategy has precedent in computational studies of battery electrolyte solvation structure and dynamics^15^.

Table S2. Table S3. Summary of DFT single-point energies for SSIP and CIP ion-pair configurations extracted from NPT-equilibrated MD trajectories at 233.15, 273.15, and 333.15 K, along with isolated EC and PF₆⁻ reference energies used for conformational transition energy calculations.

| structure | E(Hartree) | Conformational Transition Energy(Hartree) |
| --- | --- | --- |
| 233.15_SSIP | -2215.70692254569 | 0.156632104 |
| 233.15_CIP | -3069.89016938209 |  |
| ec_233.15 | -342.19925068000 |  |
| PF_6_^-^_233.15 | -940.46881549000 |  |
| 273.15_SSIP | -2215.66911448337 | 0.09375248 |
| 273.15_CIP | -2471.64162219320 |  |
| ec_273.15 | -342.20365695000 |  |
| PF_6_^-^_273.15 | -940.47357409000 |  |
| 333.15_SSIP | -2215.66328803063 | 0.074884966 |
| 333.15_CIP | -2471.64840537432 |  |
| ec_333.15 | -342.21056058000 |  |
| PF_6_^-^_333.15 | -940.48112347000 |  |

Table S3. DFT-calculated binding energies of Na⁺ in SSIP and CIP configurations, weighted average binding energies, and corresponding Na⁺ ionic mobilities from MD simulations.

| structure | FINAL SINGLE POINT ENERGY(Hartree) | Binding energy(Hartree) | Weighted Average Binding Energy(Hartree) | MD_u_Na^+^(10^-4^ cm^2^ V^-1^ s^-1^) |
| --- | --- | --- | --- | --- |
| 233.15_SSIP | -2215.706922545 | 0.115893386 | 0.115753101 | 9.8490e-06 |
| 233.15_SSIP_Na^+^ | -162.1384137051 |  |  |  |
| 233.15_SSIP_shell | -2053.452615454 |  | 20.1% CIP |  |
| 233.15_CIP | -3069.890169382 | 0.115195454 |  |  |
| 233.15_CIP_Na^+^ | -162.1384137052 |  | 79.9 %SSIP |  |
| 233.15_CIP_shell | -2907.636560223 |  |  |  |
| 273.15_SSIP | -2215.669114483 | 0.10920395 | 0.1090674 | 1.0028e-04 |
| 273.15_SSIP_Na^+^ | -162.1384137052 |  |  |  |
| 273.15_SSIP_shell | -2053.421496828 |  | 51.8 %CIP |  |
| 273.15_CIP | -2471.641622193 | 0.108940341 |  |  |
| 273.15_CIP_Na^+^ | -162.1384137052 |  | 48.2 %SSIP |  |
| 273.15_CIP_shell | -2309.394268147 |  |  |  |
| 333.15_SSIP | -2215.663288030 | 0.111112946 | 0.107687626 | 1.7972e-04 |
| 333.15_SSIP_Na^+^ | -162.1384137052 |  |  |  |
| 333.15_SSIP_shell | -2053.413761379 |  | 61.7 %CIP |  |
| 333.15_CIP | -2471.648405374 | 0.105561374 |  |  |
| 333.15_CIP_Na^+^ | -162.1384137052 |  | 38.3 %SSIP |  |
| 333.15_CIP_shell | -2309.404430294 |  |  |  |

6. Calculation of Na+ Diffusion Coefficient and Mobility Based on Mean Squared Displacement

The macroscopic transport properties of sodium ions were obtained by analyzing the Mean Squared Displacement (MSD) of trajectories from the NVT production phase. Trajectory reading and coordinate processing were performed using the MDAnalysis library^16, 17^.

Trajectory Unwrapping:Prior to MSD calculation, the trajectory coordinates of Na^+^ were unwrapped to eliminate coordinate jumps caused by periodic boundary conditions (PBC). Specifically, the minimum image convention was applied to the displacement increment Δr between consecutive frames:

Δr_unwrapped_ =Δr−L∘round( Δr/L)

where L is the simulation box length vector and ∘denotes element-wise multiplication. This procedure ensures that the continuous displacement trajectory of Na⁺ is not truncated by PBC, thereby yielding physically meaningful cumulative displacements. The first 100 frames (equilibration phase) were skipped, and the subsequent 8,000 frames (frame interval: 2.5 ps, total analysis duration: 20 ns) were used for analysis.

FFT-Based MSD Calculation:The MSD was computed using a Fast Fourier Transform (FFT) algorithm based on the Wiener-Khinchin theorem^18^, which reformulates the MSD as a frequency-domain calculation of the coordinate autocorrelation function. This reduces the computational complexity from O( N^2^ ) to O(N logN) , enabling the full utilization of all time origins for statistical averaging. For N Nasodium ions, the ensemble-averaged MSD is defined as:

$$\mathrm{MSD}(t)=\frac{1}{N_{\mathrm{Na}}}{\sum_{i=1}^{N_{\mathrm{Na}}} \left\langle\left| \mathbf{r}_{i}(t_{0}+t)-\mathbf{r}_{i}(t_{0}) \right|^{2} \right\rangle}_{t_{0}}$$

Where ⟨⋅⟩t_0_ denotes sliding-window averaging over all possible time origins t_0_.

Extraction of Diffusion Coefficient ( D ):The self-diffusion coefficient of Na⁺ was extracted via the three-dimensional Einstein relation:

$$D=\lim_{t\to\infty} \frac{\mathrm{MSD}(t)}{6t}$$

In practice, linear regression was performed over the 10%–80% interval of the total MSD time window (avoiding the initial ballistic regime and the terminal region dominated by statistical noise). The diffusion coefficient D was obtained by dividing the fitted slope by 6. The goodness of fit R^2^ exceeded 0.99 in all cases, confirming the linear characteristics of the diffusive behavior. Error estimates were obtained by calculating the standard error of the mean (SEM) from the individual MSD values of all Na⁺.

Conversion to Ionic Mobility (*μ*):Upon obtaining the self-diffusion coefficient, the absolute mobility of Na⁺ was calculated via the Nernst-Einstein equation:

$$\mu=\frac{qD}{k_{B}T}$$

where q= + e is the charge of the sodium ion, k_B_ is the Boltzmann constant, and T is the absolute temperature.

Statistical convergence of the diffusion coefficients and finite-size effects. Diffusion coefficients were obtained from ensemble-averaged MSDs with linear-fit R² above 0.99 over the 10%–80% time window, confirming well-established diffusive behavior over the production trajectory. The box edge (about 55 Å) exceeds the first-shell diameter (about 5 Å) by roughly elevenfold. For self-diffusion under periodic boundary conditions, finite-size corrections to the diffusion coefficient scale linearly with inverse box length 1/L, as described by the hydrodynamic correction formalism of Yeh and Hummer^19^. For the present system, the correction amounts to approximately 10%, far smaller than the order-of-magnitude temperature trend, and acts in the same direction at all temperatures.

**References**：

1.	Plimpton, S., Fast Parallel Algorithms for Short-Range Molecular Dynamics. *Journal of Computational Physics* **1995,** *117* (1), 1-19.

2.	Jewett, A. I.; Stelter, D.; Lambert, J.; Saladi, S. M.; Roscioni, O. M.; Ricci, M.; Autin, L.; Maritan, M.; Bashusqeh, S. M.; Keyes, T.; Dame, R. T.; Shea, J.-E.; Jensen, G. J.; Goodsell, D. S., Moltemplate: A Tool for Coarse-Grained Modeling of Complex Biological Matter and Soft Condensed Matter Physics. *Journal of Molecular Biology* **2021,** *433* (11), 166841.

3.	Dodda, L. S.; Cabeza de Vaca, I.; Tirado-Rives, J.; Jorgensen, W. L., LigParGen web server: an automatic OPLS-AA parameter generator for organic ligands. *Nucleic Acids Research* **2017,** *45* (W1), W331-W336.

4.	Jorgensen, W. L.; Tirado-Rives, J., Potential energy functions for atomic-level simulations of water and organic and biomolecular systems. *Proceedings of the National Academy of Sciences* **2005,** *102* (19), 6665-6670.

5.	Dodda, L. S.; Vilseck, J. Z.; Tirado-Rives, J.; Jorgensen, W. L., 1.14*CM1A-LBCC: Localized Bond-Charge Corrected CM1A Charges for Condensed-Phase Simulations. *The Journal of Physical Chemistry B* **2017,** *121* (15), 3864-3870.

6.	Jorgensen, W. L.; Ghahremanpour, M. M.; Saar, A.; Tirado-Rives, J., OPLS/2020 Force Field for Unsaturated Hydrocarbons, Alcohols, and Ethers. *The Journal of Physical Chemistry B* **2024,** *128* (1), 250-262.

7.	Doherty, B.; Zhong, X.; Gathiaka, S.; Li, B.; Acevedo, O., Revisiting OPLS Force Field Parameters for Ionic Liquid Simulations. *Journal of Chemical Theory and Computation* **2017,** *13* (12), 6131-6145.

8.	Lutsenko, D. S.; Belova, E. V.; Zakharkin, M. V.; Drozhzhin, O. A.; Antipov, E. V., Low-Temperature Properties of the Sodium-Ion Electrolytes Based on EC-DEC, EC-DMC, and EC-DME Binary Solvents. *Chemistry* **2023,** *5* (3), 1588-1598.

9.	Johnson, P. H., THE PROPERTIES OF ETHYLENE CARBONATE AND ITS USE IN ELECTROCHEMICAL APPLICATIONS A Literature Review. *Lawrence Berkeley National Laboratory LBNL Report : LBL-19886.*

10.	Ester, M.; Kriegel, H.-P.; Sander, J.; Xu, X., A density-based algorithm for discovering clusters in large spatial databases with noise. In *Proceedings of the Second International Conference on Knowledge Discovery and Data Mining*, AAAI Press: Portland, Oregon, 1996; pp 226–231.

11.	Neese, F., The ORCA program system. *WIREs Computational Molecular Science* **2012,** *2* (1), 73-78.

12.	Neese, F., Software update: the ORCA program system, version 4.0. *WIREs Computational Molecular Science* **2018,** *8* (1), e1327.

13.	Neese, F., Software update: The ORCA program system—Version 5.0. *WIREs Computational Molecular Science* **2022,** *12* (5), e1606.

14.	Xu, K., Nonaqueous Liquid Electrolytes for Lithium-Based Rechargeable Batteries. *Chemical Reviews* **2004,** *104* (10), 4303-4418.

15.	Jeong, H.; Wang, H.; Cheng, L., Computational prediction of solvation structures in calcium battery electrolytes. *Journal of Materials Chemistry A* **2024,** *12* (47), 33150-33161.

16.	Michaud-Agrawal, N.; Denning, E. J.; Woolf, T. B.; Beckstein, O., MDAnalysis: A toolkit for the analysis of molecular dynamics simulations. *Journal of Computational Chemistry* **2011,** *32* (10), 2319-2327.

17.	Gowers, R. J.; Linke, M.; Barnoud, J.; Reddy, T. J. E.; Melo, M. N.; Seyler, S. L.; Domański, J.; Dotson, D. L.; Buchoux, S.; Kenney, I. M.; Beckstein, O. In *MDAnalysis: A Python Package for the Rapid Analysis of Molecular Dynamics Simulations*, Proceedings of the 15th Python in Science Conference, 2016; 2016.

18.	Calandrini, V.; Pellegrini, E.; Calligari, P.; Hinsen, K.; Kneller, G. R., nMoldyn - Interfacing spectroscopic experiments, molecular dynamics simulations and models for time correlation functions. *École thématique de la Société Française de la Neutronique* **2011,** *12*, 201-232.

19.	Yeh, I.-C.; Hummer, G., System-Size Dependence of Diffusion Coefficients and Viscosities from Molecular Dynamics Simulations with Periodic Boundary Conditions. *The Journal of Physical Chemistry B* **2004,** *108* (40), 15873-15879.
